# Effect of Participative Web-Based Educational Modules on HIV and Sexually Transmitted Infection Prevention Competency Among Medical Students: Single-Arm Interventional Study

**DOI:** 10.2196/42197

**Published:** 2023-01-24

**Authors:** William Grant, Matthew A Adan, Christina A Samurkas, Daniela Quigee, Jorge Benitez, Brett Gray, Caroline Carnevale, Rachel J Gordon, Delivette Castor, Jason Zucker, Magdalena E Sobieszczyk

**Affiliations:** 1 Duke University School of Medicine Duke University Durham, NC United States; 2 Vagelos College of Physicians & Surgeons Columbia University New York, NY United States; 3 Division of Infectious Diseases Department of Internal Medicine Columbia University Irving Medical Center New York, NY United States

**Keywords:** HIV prevention, medical education, sexual health education, pre-exposure prophylaxis, PrEP

## Abstract

**Background:**

The number of new HIV diagnoses in the United States continues to slowly decline; yet, transgender women and men who have sex with men remain disproportionately affected. Key to improving the quality of prevention services are providers who are comfortable broaching the subjects of sexual health and HIV prevention with people across the spectrum of gender identities and sexual orientations. Preservice training is a critical point to establish HIV prevention and sexual health education practices before providers’ practice habits are established.

**Objective:**

The study aimed to develop participative web-based educational modules and test their impact on HIV prevention knowledge and awareness in future providers.

**Methods:**

Sexual health providers at an academic hospital, research clinicians, community engagement professionals, and New York City community members were consulted to develop 7 web-based educational modules, which were then piloted among medical students. We assessed knowledge of HIV and sexually transmitted infection prevention and comfort assessing the prevention needs of various patients via web-based questionnaires administered before and after our educational intervention. We conducted exploratory factor analysis of the items in the questionnaire.

**Results:**

Pre- and postmodule surveys were completed by 125 students and 89 students, respectively, from all 4 years of training. Before the intervention, the majority of students had heard of HIV pre-exposure prophylaxis (122/123, 99.2%) and postexposure prophylaxis (114/123, 92.7%). Before the training, 30.9% (38/123) of the students agreed that they could confidently identify a patient who is a candidate for pre-exposure prophylaxis or postexposure prophylaxis; this increased to 91% (81/89) after the intervention.

**Conclusions:**

Our findings highlight a need for increased HIV and sexually transmitted infection prevention training in medical school curricula to enable future providers to identify and care for diverse at-risk populations. Participative web-based modules offer an effective way to teach these concepts.

## Introduction

### Background

Since 2017, the overall rate of new HIV diagnoses in the United States has declined each year owing to HIV testing, treatment as prevention, and advances in biomedical prevention such as pre-exposure prophylaxis (PrEP) and postexposure prophylaxis (PEP). However, transgender women and men who have sex with men are disproportionately represented in new HIV diagnoses each year [1,2]. The reasons for these disparities are multifactorial, but key to improving access to, and quality of, HIV prevention services are knowledgeable providers who are comfortable addressing topics of HIV prevention and sexual health concerns across gender identities, sexual orientations, and age. Providers frequently serve as key facilitators to accessing prevention services. Focus group meetings among lesbian, gay, bisexual, transgender, and queer (LGBTQ) individuals conducted previously by our group identified that an important factor in accessing prevention services and participating in HIV prevention research studies was receiving information from providers experienced in providing care to gender-diverse individuals [3]. When LGBTQ individuals such as minoritized Black men who have sex with men are stigmatized by health care providers, this leads to distrust of providers, lack of sexual orientation disclosure, delays in seeking needed medical care, and incomplete disclosure of risk-taking behaviors related to HIV [4-10]. A survey of 120 American internal medicine residents revealed that only 2.3% had ever prescribed PrEP, with the top barrier being lack of familiarity, likely because of a lack of provider education and training [11]. Discomfort with sexual history taking and genital examinations was identified as a barrier to sexually transmitted infection (STI) testing [12,13] and decreased the likelihood of prescribing PrEP [14-16].

### Objectives

Education can change providers’ intentions and practices [17]. We propose that for lasting impact, it is important to start HIV prevention and sexual health education before inadequate practice habits are firmly established. Therefore, medical students are an important group to train to shape future HIV prevention practices and knowledge. Data about knowledge of, and attitudes toward, HIV prevention among medical students are fairly limited but reveal concerns about inadequate preparation for future practice [18-20]. A recent survey of medical students found that only 37.6% felt adequately trained to address sexual health concerns of patients, and other surveys revealed that students do not feel fully prepared to care for LGBTQ patients [21-24]. Focused training on HIV prevention, gender identity, and sexual orientation and behaviors provided early in medical education may remove barriers and stigmatization for LGBTQ patients. We proposed to address this need by creating participative educational modules adapted for medical students. Novel approaches such as web-based platforms that permit participative learning, incorporate feedback, and use role-playing have proven extremely successful when used by infectious diseases faculty at an academic medical center to teach medical students general infectious diseases and virology [25]. This investigation built on the expertise of the research team to create participative modules that focus on topics of HIV prevention, sexual health, risk reduction, and the biomedical prevention research pipeline. We tested the impact of these modules on knowledge of STI and HIV testing as well as PEP and PrEP in a cohort of first- through fourth-year medical students.

We hypothesized that participative web-based modules would increase medical students’ knowledge of PrEP and PEP, increase confidence in identifying candidates for HIV prevention services, and serve as acceptable learning tools for medical students.

## Methods

### Primary Outcome Measures

Our main outcome measures were student-reported comfort and confidence in engaging with LGBTQ patients, student-reported sexual history–taking abilities, and confidence in identifying patients who are candidates for PrEP and PEP (5-point Likert scale). We also assessed general knowledge of HIV and STI screening and prevention (10-point scale).

### Module Development

The educational modules were developed between September 2018 and January 2019 using *Articulate Storyline* (Articulate Global, LLC). Sexual health providers, research clinicians, and community engagement volunteers at a large urban tertiary care academic medical center located in a predominantly Latinx (72%) and foreign-born (47%) community in New York City were consulted for expertise and supplemental materials on risk reduction counseling, prescribing, and monitoring patients on PrEP and PEP, as well as biomedical prevention research studies [26]. These materials were used to develop unique clinical narratives and cases that were web based and participative. The finalized module content is presented in Textbox 1.

After initial drafts of the modules were constructed, the same sexual health providers, research clinicians, and community engagement volunteers who were consulted before module creation were asked to offer feedback on content accuracy, language, and organization. The modules were hosted on a web-based secure server established by the research team. These modules can be viewed at Stick2PrEP [27].

**Finalized module content.**

**Seven 5- to 10-minute modules**

1. A postexposure prophylaxis (PEP) module on the indications and evidence behind PEP and how to monitor a patient on PEP
2. PEP cases where students engaged with 4 distinct clinical cases based on the foundational knowledge and skills learned in the PEP module
3. A pre-exposure prophylaxis (PrEP) module on laboratory testing, prescribing, and clinical indications for PrEP
4. PrEP cases where students applied the knowledge learned in the PrEP module by navigating 4 patient cases
5. A sexually transmitted infection testing module focused on special considerations when screening and treating diverse patient populations such as cisgender men who have sex with men, geriatric populations, patients living with HIV or AIDS, and transgender women
6. A sexual health algorithm about the appropriate terminology to use when interacting with gender and sexually diverse patients, creating a welcoming environment for lesbian, gay, bisexual, transgender, or queer patients, and gendered pronoun use, with concepts supplemented by 2 clinical cases
7. Research concepts that explored HIV prevention in the research setting, such as preventive vaccine and antibody studies, topical microbicides, and long-acting injectable PrEP

### Advisory Group

Community members aged ≥18 years who lived in the New York City metropolitan area and had seen a provider more than once in the last 12 months for unspecified medical reasons were invited to provide contact information to participate in a community advisory group about their HIV and STI testing experiences and provide feedback on initial versions of the educational modules. Gender and sexual minorities were strongly encouraged to participate. Community members were recruited via Craigslist, Facebook, and physical flyers posted on the medical center campus. Of the 116 eligible community members who responded to the advertisements, up to 16 (13.8%) were contacted for each advisory group, with gender identity, sexual orientation, risk factors for HIV and STI infection, and clinical experiences being relevant to the selection process. After we obtained informed consent from all participants, 2 advisory group meetings were conducted in November 2018. Two members of the research team, MAA and WG, facilitated these meetings. All community members were reimbursed US $25 for their time and thoughtful contributions. These advisory group meetings followed a prepared script, and audio recordings of both meetings were transcribed. Two research team members identified reoccurring themes from the transcripts, which were then used to further inform the content of the modules. Two iterations of the modules based on advisory group feedback occurred, incorporating feedback from the first group (iteration 1) and the second group (iteration 2).

### Medical Student Questionnaires

We used 20 items to assess student confidence, knowledge, and perception of sexual health, which were assessed before and after completion of the educational modules. To our knowledge, no validated survey instruments exist to measure these concepts. Thus, the survey instrument was developed based on a review of published literature and clinical experience of the investigative team. Question content and phrasing were developed collaboratively by authors WG, MAA, CC, JZ, and MEK. The remaining members of the research team offered feedback on an initial draft of the questionnaire. The questions used in the assessment are presented in Textbox 2. The first 10 questions were assessed on a 5-point Likert scale, ranging from 1=*strongly disagree* to 5=*strongly agree*. The next set of 10 questions, based on HIV and STI screening and prevention knowledge, was presented in a multiple-choice format and graded for correctness on a scale of 0 to 10, with each question weighted equally. The students were asked to provide demographic information to capture relevant educational and social variables (ie, age, gender, race, sexual orientation, and familiarity with PrEP and PEP).

**Medical student questionnaire. LGBTQ: lesbian, gay, bisexual, transgender, and queer; PEP: postexposure prophylaxis; PrEP: pre-exposure prophylaxis; STI: sexually transmitted infection.**

**Likert-scale questions: comfort with taking a sexual history and with sexual and gender minorities, as well as identifying candidates for postexposure prophylaxis and pre-exposure prophylaxis (questions 6, 7, 8, and 9 were removed from the pre- vs postintervention analysis based on factor structure determined via exploratory factory analysis)**

1. I feel comfortable asking patients about their sexual orientation e.g. gay, bisexual.
2. I feel comfortable discussing sexual health problems with patients of different gender identity than my own.
3. I feel comfortable taking a sexual history from a patient who identifies as LGBTQ.
4. I feel comfortable asking patients about their sexual practices e.g. “Are you sexually active?”, “Do you practice vaginal sex?”
5. I find taking a sexual history easy.
6. I have adequate skills to take a sexual history.
7. I have enough exposure as a medical student to take a sexual history from a real patient.
8. I have enough exposure as a medical student to take a sexual history from a simulated patient.
9. I feel that there is not enough training in medical school on how to discuss sexual health problems with patients.
10. I feel confident identifying a patient who is a candidate for PrEP, PEP, and other HIV and STI prevention services.

**Multiple-choice questions: HIV and sexually transmitted infection screening and prevention knowledge (for the multiple-choice questions, students were presented with 4 options not shown here; they did not receive correct-response feedback)**

1. How often should all sexually active gay, bisexual, and other men who have sex with men (MSM) be screened for HIV, syphilis, chlamydia, and gonorrhea?
2. According to the CDC, annual chlamydia screening is recommended for all sexually active women under the age of ___, as well as older women with risk factors such as ___.
3. 4th generation HIV tests detect ___ in blood specimens.
4. PrEP, when used daily and with condoms, has been shown to reduce the risk of HIV infection in those who are high risk by up to___.
5. Which of these individuals would benefit from PrEP use?
6. At time of initiation of a PrEP regimen, how many days of medication should you prescribe at the first patient visit?
7. How many days of medication should you prescribe at an initial PEP visit?
8. How many hours after HIV exposure should PEP be started?
9. Which of these individuals would be a candidate for PEP (assume within appropriate time window)?
10. True or False: HIV negative recipients of an HIV vaccine may test positive on some HIV antibody tests for the duration of a vaccine study and possibly thereafter.

Completion of the survey was anonymous and not time restricted. The questionnaire was administered using Qualtrics survey software and was open only to medical students at the institution where the modules were developed. Informed consent was obtained using the cover page of the survey. Electronic invitations to participate in the survey were distributed using class listserve accounts. Administrative permission was obtained before sending invitations to student listserve accounts. Participant eligibility and inclusion criteria were defined as currently enrolled first- through fourth-year medical students. Medical students at the recruitment site (approximately 150 per class) participate in a 4-year curriculum, with full-time classroom-based teaching for the first 1.5 years (3 semesters) of the curriculum, after which they begin their clinical rotations. Exposure to HIV and STI testing occurs during the infectious diseases unit in the third semester and as is relevant during clinical rotations. Some fourth-year students were recruited for participation via a month-long residency preparedness course taken just before the intern year. The questionnaire and participation were offered as voluntary supplemental learning opportunities.

After completion of the premodule survey, the students were routed to another Qualtrics survey whereby they could provide an email address to receive a URL link to the learning modules. Students were given up to 2 weeks to complete the 7 learning modules to facilitate focused learning and to allow knowledge gained from one module to be applied to the next. At the end of the final module, participants received a link to complete an anonymous postmodule Qualtrics survey.

The postmodule questionnaire was used to assess the same domains included in the premodule questionnaire and used the same 20-item assessment. It also contained a space for free-text entry to provide general thoughts and comments on the modules. However, the postmodule questionnaire did not include demographic information in an effort to maintain student anonymity. For this reason, the pre- and posttest surveys could not be linked at the individual level. Local institutional review board approval was obtained before starting the study (refer to the *Ethics Approval* section), and all methods were performed in accordance with the Declaration of Helsinki. Grant funding was used to reimburse students US $50 for completing the modules.

### Statistical Analysis

Exploratory factor analysis (EFA) was conducted to investigate the factor structure of the Likert-scale questions of the questionnaire. As a first step, parallel analysis, minimum average partial, and a scree plot were used to determine the number of factors to extract for the EFA. Subsequently, several models with different numbers of factors, suggested by the initial analysis, were fitted via weighted least squares (WLS). We anticipated that the underlying factors were intercorrelated. Therefore, oblimin and promax oblique rotations were used and their results compared.

Each model was evaluated by examining whether it exhibited salient pattern loadings (loadings ≥0.32), showed an approximate simple structure, and contained considerable intercorrelations among the factors. A root mean squared residual (RMSR) of ≤0.08 was considered an acceptable model fit. The proportion of residual coefficients that exceeded absolute values of 0.05 and 0.10 were also examined. Finally, the Cronbach α reliability coefficient for each subscale had to approach a value of.90 for a model to be deemed acceptable.

For the resulting factor model, median scores with IQRs were calculated both before and after the intervention. *P* values for comparing pre- and postmodule responses were determined using Wilcoxon rank sum tests. For HIV and STI screening and prevention knowledge, percentage correct was calculated for each question, and *P* values were determined using the 2-sample binominal test for proportions using normal theory methods with continuity correction. *P* values were Bonferroni corrected. Median HIV and STI screening and prevention knowledge scores were compared via Wilcoxon rank sum tests. Given the paired nature of the data, we intended to use Wilcoxon signed-rank tests; however, without means of linking the premodule and postmodule questionnaire responses, the individual-level data could not be paired. The purpose of keeping the premodule and postmodule responses unlinked was to maintain the anonymity of the students in accordance with the institutional review board protocol. All data were analyzed using RStudio 2022.02.2+485 *Prairie Trillium* release (Posit Software, PBC) and Microsoft Excel (version 16.62).

### Ethics Approval

This investigation was conducted in accordance with the Declaration of Helsinki and was approved by the institutional review board at Columbia University Irving Medical Center (AAAR8304). Informed consent was obtained from all medical student participants via the premodule web-based questionnaire and from all community members who participated in the advisory groups.

## Results

### Survey Response and Demographics

**Figure 1:**
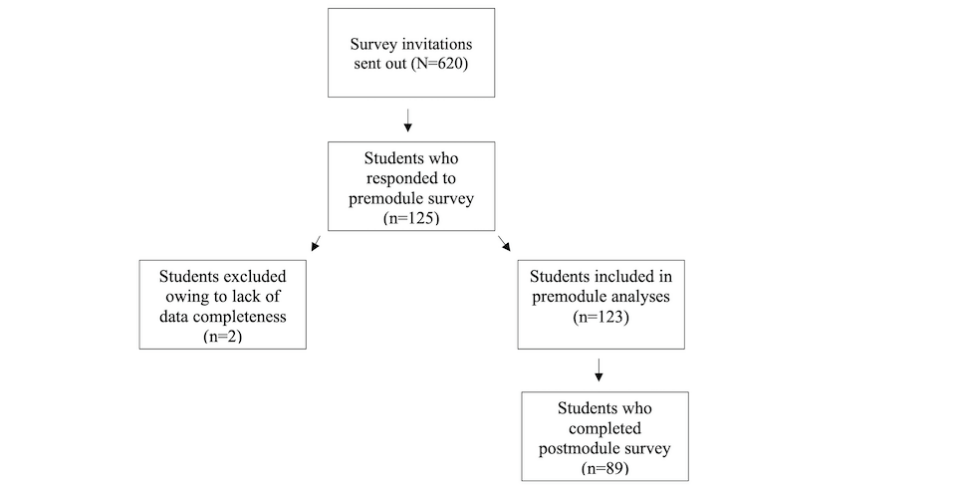
Flowchart of study participation and completion.

A total of 620 survey invitations were sent to medical students via email or the institution offering the residency preparation course; we received responses from 125 individuals, representing a 20.2% response rate. Two responses were excluded from data analyses owing to lack of data completeness. The mean age of the 123 students in the final sample was 26.5 (SD 2.4) years, and fourth-year students were most represented among all student cohorts (51/123, 41.5%). The majority of students identified as White (62/123, 50.4%), heterosexual (96/123, 78.1%), and women (71/123, 57.5%), whereas 22% (27/123) identified as lesbian, gay, bisexual, or other or did not provide a response. Most students had heard of PrEP and PEP before the educational modules (122/123, 99.2%, and 114/123, 92.7%, respectively). Complete participant characteristics are summarized in Table 1. A total of 89 students also completed a postmodule survey. The overall completion rate was 71.2% (89/125). Figure 1 summarizes study participation and completion.

**Table 1:** Baseline medical student characteristics, demographic information, and questionnaire scores.

						M1^a^ (n=12)		M2 (n=37)		M3 (n=23)		M4 and M4+^b^ (n=51)		Total (N=123)
**Demographic characteristics**														
	Age (years), mean (SD)					24.9 (2.6)		25.3 (2.4)		27.1 (1.5)		27.5 (2.1)		26.5 (2.4)
	**Gender identity^c^, n (%)**													
				Man		6 (50)		15 (40.5)		8 (34.8)		22 (43.1)		51 (41.5)
				Woman		6 (50)		21 (56.8)		15 (65.2)		29 (56.9)		71 (57.7)
				Nonbinary		0 (0)		1 (2.7)		0 (0)		0 (0)		1 (0.8)
	**Race^d^, n (%)**													
				Black, non-Hispanic		0 (0)		4 (10.8)		2 (8.7)		4 (7.8)		10 (8.1)
				White, non-Hispanic		7 (58.3)		16 (43.2)		12 (52.3)		27 (52.9)		62 (50.4)
				Asian or Pacific Islander, non-Hispanic		2 (16.7)		12 (32.4)		5 (21.7)		9 (17.6)		28 (22.8)
				Hispanic or Latinx		2 (16.7)		3 (8.1)		3 (13)		1 (2)		9 (7.3)
				Mixed race or other		1 (8.3)		2 (5.4)		1 (4.3)		10 (19.6)		14 (11.4)
	**Sexual orientation, n (%)**													
				Lesbian		0 (0)		1 (2.7)		0 (0)		1 (2)		2 (1.6)
				Gay		2 (16.7)		2 (5.4)		2 (8.7)		7 (13.7)		13 (10.6)
				Bisexual		1 (8.3)		3 (8.1)		0 (0)		4 (7.8)		8 (6.5)
				Heterosexual		8 (66.7)		29 (78.4)		20 (87)		39 (76.5)		96 (78.1)
				Other or no response		1 (8.3)		2 (5.4)		1 (4.3)		0 (0)		4 (3.3)
	Heard of PrEP^e^, n (%)					12 (100)		36 (97.3)		23 (100)		51 (100)		122 (99.2)
	Heard of PEP^f^, n (%)					10 (83.3)		34 (91.9)		23 (100)		47 (92.2)		114 (92.7)
	**Confidence identifying candidates for PEP and PrEP, n (%)**													
			Strongly agree		1 (8.3)		3 (8.1)		2 (8.7)		5 (9.8)		11 (8.9)	
			Agree		3 (25)		8 (21.6)		6 (26.1)		10 (19.6)		27 (22)	
**Questionnaire scores, median (IQR)**														
		Factor 1^g^				4.0 (3.0-4.0)		3.0 (3.0-4.0)		4.0 (3.0-4.0)		4.0 (3.0-4.0)		4.0 (3.0-4.0)
		HIV and STI^h^ screening and prevention^i^				7.0 (6.0-7.0)		6.0 (5.0-7.0)		6.0 (6.0-8.0)		7.0 (5.0-8.0)		(6.0-7.0)

^a^M1, M2, M3, and M4: year of medical education.

^b^M4+: students who have completed >4 years of medical training (ie, dual degree or research year).

^c^Students were given the option of selecting multiple gender identities. Transgender (female to male), transgender (male to female), and unlisted term with free-text option were aggregated into *Other*.

^d^Students who selected multiple racial categories were grouped into *Mixed race or other*.

^d^PrEP: pre-exposure prophylaxis.

^f^PEP: postexposure prophylaxis.

^g^Assessed on a Likert scale of 1 to 5.

^h^STI: sexually transmitted infection.

^i^Assessed on a scale of 0 to 10, based on the number of questions answered correctly.

### Measurement Psychometrics

Of the 10 Likert-scale questions presented in Textbox 2, question 9 was removed from the analysis because it did not correlate with any other question (no Pearson *r* values >0.3) and had the lowest item-total correlation (*r*=–0.17); hence, it would not have contributed meaningfully to the analysis. The initial analysis using the previously described factor extraction methods and incorporating the remaining 9 questions suggested a 1- to 2-factor model. A 2-factor model was most appropriate (RMSR=0.034) but had increased complexity resulting from question 6 loading almost equally on both factors (complexity=1.97, WLS1=0.465, WLS2=0.413). Upon further inspection, the wording of question 6 was noted to be highly similar to that of question 5; therefore, question 6 was removed too. In subsequent models with 8 questions included, questions 1, 2, 3, 4, 5, and 10 loaded on the first factor, whereas questions 7 and 8 loaded on the second factor. Given that any factor should comprise at least 3 contributing questions, the 2 questions loading on the second factor (questions 7 and 8) were removed from the analysis [28]. In sum, of the 10 Likert-scale questions, 6 were deemed appropriate for inclusion in the pre- to postintervention statistical analysis. Those removed are noted in Textbox 2.

Parallel analysis, the scree plot, empirical scree tests, and the minimum average partial all suggested an EFA with a single factor, henceforth referred to as factor 1. The RMSR for the resulting single factor model was 0.041, which is below the a priori cutoff of 0.08. Factor loadings for the 6 questions that comprise factor 1 ranged from 0.490 to 0.799. In this model, there were no residuals >0.10 and only 27% >0.05. The Cronbach α value for factor 1 was .87 (95% CI 0.82-0.90), and reliability did not increase when any individual factor was dropped, thus supporting the 1-factor structure and inclusion of these 6 questions.

### Pre- to Postintervention Analysis

**Figure 2:**
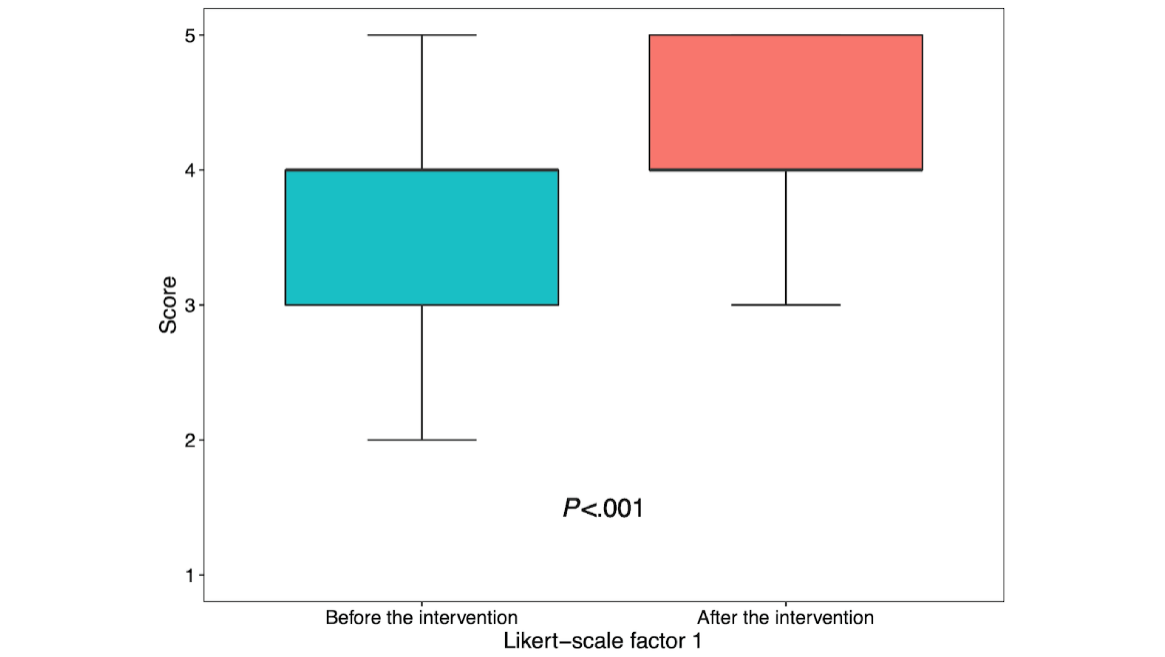
Factor 1 before and after the educational intervention (premodule survey: n=123 and postmodule survey: n=89). Data are shown as box-and-whisker plots with the lower and upper limits (bounds) of the box representing quartile 1 (25th percentile) and quartile 3 (75th percentile), respectively. The median (quartile 2, 50th percentile) is represented by the bolded horizontal line within each box. Whiskers, shown as vertical lines extending from the boxes, extend to 1.5 times the IQR. IQR: interquartile range.

For factor 1, although the median score did not change, the IQR increased, given a median of 4.0 (IQR 3.0-4.0) before the intervention and 4.0 (IQR 4.0-5.0) after the intervention (*P*<.001; Figure 2). The frequency of the score of 5 (*strongly agree*) increased from 15% to 35%. Specifically for confidence identifying a candidate for PEP or PrEP, the median score increased from 3.0 (IQR 2.0-4.0) to 4.0 (IQR 4.0-5.0; *P*<.001). The frequency of the score of 4 (*agree*) increased from 22% to 53%, and the frequency of the score of 5 (*strongly agree*) increased from 9% to 38%. These data are summarized in Multimedia Appendix 1.

Although 4 questions were removed from the factor analysis, some of these questions demonstrated statistically significant increases from before to after the intervention; for example, when asked to rate agreement with question 7 (“I have enough exposure as a medical student to take a sexual history from a real patient”), the median score increased from 3.0 (IQR 2.0-4.0) to 4.0 (IQR 3.0-4.0; *P*=.02). Agreement with question 6 (“I have adequate skills to take a sexual history”) also increased from 4.0 (IQR 3.0-4.0) to 4.0 (IQR 4.0-5.0; *P*<.001).

**Figure 3:**
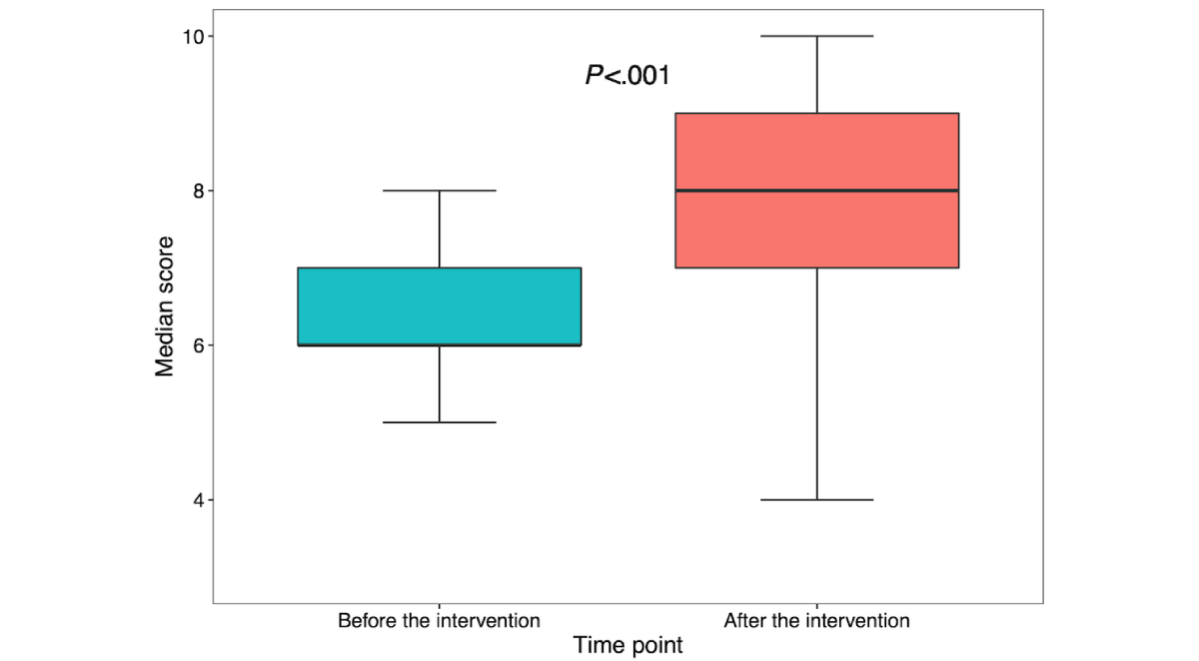
Pre- and posteducational intervention HIV and sexually transmitted infection screening and prevention knowledge (premodule median scores: n=123 and postmodule median scores: n=89). HIV and sexually transmitted infection screening and prevention knowledge scores are on a scale of 0 to 10 and represent general sexual health screening and prevention questions scored for correctness. Data are shown as box-and-whisker plots with the lower and upper limits (bounds) of the box representing quartile 1 (25th percentile) and quartile 3 (75th percentile), respectively. The median (quartile 2, 50th percentile) is represented by the bolded horizontal line within each box. Whiskers, shown as vertical lines extending from the boxes, extend to 1.5 times the IQR.

The median HIV and STI screening and prevention knowledge score also increased from a baseline of 6.0 (IQR 6.0-7.0) to 8.0 (IQR 7.0-9.0; *P*<.001; Figure 3). Pre- to postintervention changes in the scores for the 10 individual questions on HIV and STI screening and prevention knowledge are summarized in Table 2; the questions are presented in Textbox 2. Although there was an increase in the percentage of correct responses for all questions after the educational intervention, 4 of the 10 questions met our criteria for statistical significance (*P*<.005 after Bonferroni correction). All statistically significant changes in correct responses involved prescribing, monitoring, and evidence behind PrEP and PEP. This perhaps reflects a collective gap in knowledge within this clinical domain as well as a substantial increase in knowledge of this subject after the intervention.

**Table 2:** HIV and sexually transmitted infection screening and prevention knowledge percentage of correct answers by question.

	Preintervention survey (% correct), n=123	Postintervention survey (% correct), n=89	*P* value^a^
Q1	87.8	92.1	.21
Q2	95.1	95.5	.99
Q3	68.3	77.5	.09
Q4	30.1	59.6	<.001
Q5	65.9	68.5	.40
Q6	39	73	<.001
Q7	25.2	66.3	<.001
Q8	59.3	94.4	<.001
Q9	82.1	87.6	.18
Q10	91.9	92.1	.57
Median	60	80	<.001

^a^Threshold for significance after Bonferroni correction: <.005.

### Narrative Feedback

Narrative feedback from medical students, collected as free-text entry within the postmodule survey, was overwhelmingly positive. A student stated as follows:

Great modules. This is the first time in my medical school program to learn about PEP, as well as my first formal education module on PrEP. Keep it up and make it more available to future healthcare providers.

Participant 1

Another student provided the following feedback:

Really useful modules, especially the PEP module as I received no education on post-exposure prophylaxis, as well as how to prescribe it to my patients throughout the entirety of medical school. These modules should become an integral part of our clinical training.

Participant 2

A third student stated as follows:

This was great learning. I wish it was integrated into the medical curriculum.

Participant 3

Several individuals commented that the modules were the appropriate length and that they provided useful information even for those already familiar with PrEP and PEP.

### Advisory Groups

Regarding the advisory group meetings, of the 6 community members, 2 (33%) attended the first meeting, and 4 (67%) attended the second. The first and second advisory group meetings lasted 90 minutes and 120 minutes, respectively. Demographic characteristics of the advisory group participants are summarized in Table 3. Three key themes were identified from the meetings, which were used to inform module content and are summarized in Table 4, with supporting quotations (the quotations were selected, verbatim, from audio-recorded transcripts; language was not abridged or manipulated; and transcription was performed by Transcripts 4 North America). In addition, prompted by the advisory meetings, we modified module content language to further enhance inclusivity and reorganized the workflow of several modules to improve clarity.

**Table 3:** Advisory group participant demographic information.

Advisory group meeting	Age of participant (years)	Race	Gender identity^a^	Sexual orientation	Heard of PEP^b^	Ever taken PEP	Heard of PrEP^c^	Ever taken PrEP
1	31	Mixed race (did not specify)	Woman	Heterosexual	Yes	No	Yes	No
1	24	Black, non-Hispanic	Man	Heterosexual	No	No	Yes	No
2	37	Black, non-Hispanic	Man	Gay	Yes	No	Yes	Yes
2	56	Black, non-Hispanic	Man	Bisexual	Yes	Yes	Yes	Yes
2	—^d^	White, non-Hispanic	Woman	Heterosexual	Yes	No	Yes	No
2	40	Black, non-Hispanic	Man	Heterosexual	Yes	Yes	No	No

^a^Woman refers to cisgender woman and Man refers to cisgender man. There were no participants identifying as transgender in either advisory group.

^b^PEP: postexposure prophylaxis.

^c^PrEP: pre-exposure prophylaxis.

^d^Participant did not provide response within free-text response box.

**Table 4:** Advisory group themes with supporting quotations from participants.

Themes	Illustrative quotes
Bias and stereotype in patient-provider interactions	“But maybe to not use—I don’t necessarily feel like you have to speak to minorities, gay men, or people who live in maybe impoverished neighborhoods like we are high risk just because of those factors.” [Participant 1]
Diversifying standard clinical practices	“I think they should have like a checklist of things, you know. I’ve never been to a primary care doctor that—maybe I filled it out on paper—that asked me if I’m bisexual, if I’m heterosexual, whatever. I’ve never really experienced that before.” [Participant 2]
Openly promoting access to innovative prevention services	“You know, you don’t see signs in the office that says PrEP or anything like that. You go to these community-based places and you see PrEP everywhere, you know? But you don’t see it in no primary care doc, you know, about that.” [Participant 3]

## Discussion

### Principal Findings

This study evaluated medical students’ knowledge and confidence regarding HIV and STI prevention concepts across the spectrum of gender identity and sexual orientation. Our findings suggest that there is a need for increased HIV and STI prevention training in standard medical school curricula, particularly given the recent Centers for Disease Control and Prevention recommendation that all sexually active adolescents and adults should be informed by their providers about PrEP [29]. This conclusion is supported by our findings that although most of the students had heard of PrEP (122/123, 99.2%) and PEP (114/123, 92.7%), only 30.9% (38/123) felt confident identifying patients who were candidates for these prevention therapies. Before the intervention, relatively few students could identify the number of days of medication that should be prescribed at an initial visit for PrEP (48/123, 39%) and PEP (31/123, 25.2%). Others have demonstrated that both web-based and in-person educational curricula can effectively teach sexual history taking and increase confidence in working with LGBTQ patients among first- and second-year medical students, but they did not include students in later years of medical education in these interventions [30-33]. Our study found that HIV and STI prevention knowledge was similar across years of medical education. Fourth-year medical students preparing to begin residency did not feel more confident than their juniors at identifying candidates for prevention services; nor did they report the highest confidence in their perception of their sexual history–taking abilities or confidence in interacting with LGBTQ patients. This highlights a lack of effective curricula for medical students related to sexual health and emphasizes the need for this content to not only be taught early in medical school but also be reiterated in the final years of medical education.

Many prior studies have used interventions that require in-person sessions or web-based group meetings, whereas this study demonstrates that completely self-paced web-based educational modules are an effective and easy-to-implement method of increasing medical student knowledge [30-35]; for example, the percentage of students who felt confident in identifying a candidate for prevention services increased by 60%—from 30.9% (38/123) to 91% (81/89)—after completion of the educational modules. In addition, comfort providing sexual health care to LGBTQ individuals and perception of sexual history–taking abilities, both of which are encompassed in factor 1, increased after the intervention. These findings support the use of innovative educational modules as practical and accessible learning tools to increase medical students’ knowledge.

The students’ free-text comments from the postmodule survey demonstrated that the modules were well received by participants and were viewed as an important addition to their medical education. Their comments underscored that this content was not covered elsewhere in their education and affirmed that there is a need for increased HIV and STI prevention training in standard medical school curricula. Given the positive feedback and interest from the students, these modules have now been incorporated into the second- and fourth-year medical student curricula at the institution where they were developed.

### Strengths and Limitations

This study includes several strengths. The educational modules were designed in part by sexual health clinicians who provided clinical expertise, with subsequent refinement via input from diverse community members. The use of EFA allowed for progress toward a validated instrument to measure medical student confidence in taking a sexual history and working with LGBTQ patients. The self-paced web-based nature of the modules is also a great strength of this study because it allowed for students to flexibly engage with this content at times that were most suitable for them in terms of the learning experience.

Our study is not without limitations. Pre- and postmodule questionnaires were completed anonymously, and we did not provide students with a study-specific ID or linking identifier between the pre- and postintervention responses. This limited our ability to make statistical inferences from our analyses, which had a pre-post paired design. Instead, unpaired aggregated differences were generated through our analyses. The study may have limited generalizability because the baseline characteristics of the students who completed the study do not necessarily reflect the characteristics of medical students or providers throughout the region or nationally; for example, 22% (27/123) of the students who completed the premodule survey identified as lesbian, gay, bisexual, or other in terms of sexual orientation, which is above the estimated average for the US adult population (4.5%) [36]. Some students may also have learned about HIV prevention, PrEP, and PEP through public health campaigns and other external sources in New York City; in other words, their knowledge may not be attributable to the educational modules. In addition, given that the data were gathered by self-report, it is possible that the students provided socially desirable responses and misestimated their own abilities during survey completion. If our recruitment attracted students with specific social or educational variables, this may have been a confounding element; for example, participants were not recruited in equal numbers across all years of medical school. Some students may have been drawn to the study owing to monetary compensation and may not have meaningfully engaged with the content before completing the postmodule survey. We also recognize that this analysis is exploratory in nature. We hope to repeat this study with a larger sample size and additional postmodule survey time points to further validate the survey instrument, perform a confirmatory factor analysis, and demonstrate long-term knowledge retention after module completion.

We demonstrated that web-based educational modules on the subject of HIV prevention are easy to design and implement, are viewed favorably by learners, and effectively increase medical students’ knowledge of STI testing, HIV prevention strategies, and confidence in taking a sexual history. Broader implementation of such modules in medical school curricula could enhance HIV prevention services offered by the next generation of medical providers.

## Appendix

Multimedia Appendix 1 Pre- and posteducational intervention pre-exposure prophylaxis (PrEP) and postexposure prophylaxis (PEP) confidence. (A) Pre-educational intervention PrEP and PEP confidence: n=123. Participants were asked to rate their agreement with the statement “I feel confident identifying candidates for PrEP and PEP.” (B) Posteducational intervention PrEP and PEP confidence: n=89. Participants were asked to rate their agreement with the statement “I feel confident identifying candidates for PrEP and PEP.”.

## Abbreviations

EFA: exploratory factor analysis
LGBTQ: lesbian, gay, bisexual, transgender, and queer
PEP: postexposure prophylaxis
PrEP: pre-exposure prophylaxis
RMSR: root mean squared residual
STI: sexually transmitted infection
WLS: weighted least squares

## Data Availability

The data sets generated and analyzed during this study are not publicly available because they are protected by the institutional review board at Columbia University Irving Medical Center but are available from the corresponding author on reasonable request.
