# Supplementary figures and images for: Effect of Participative Web-Based Educational Modules on HIV and Sexually Transmitted Infection Prevention Competency Among Medical Students: Single-Arm Interventional Study
Source: JMIR Med Educ. 2023 Jan 24;9:e42197. doi: 10.2196/42197 (PMC9906317; doi:10.2196/42197)

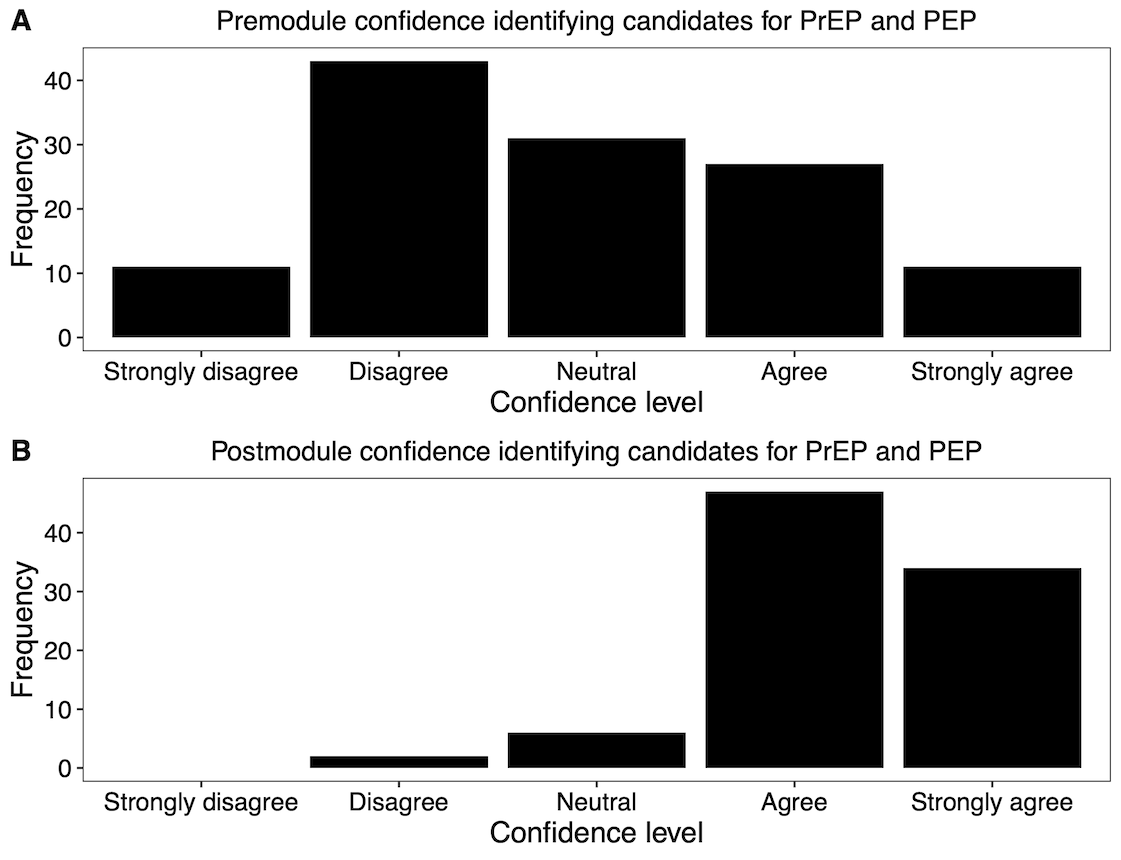

Supplement: Multimedia Appendix 1 [file mededu_v9i1e42197_app1.png]
